# Phenotypic Plasticity of HSP70s Gene Expression during Diapause: Signs of Evolutionary Responses to Cold Stress among Soybean Pod Borer Populations (*Leguminivora glycinivorella*) in Northeast of China

**DOI:** 10.1371/journal.pone.0109465

**Published:** 2014-10-15

**Authors:** Ling Wang, Shuai Yang, Lanlan Han, Dong Fan, Kuijun Zhao

**Affiliations:** College of Agriculture, Northeast Agricultural University, Harbin, China; St. Georges University of London, United Kingdom

## Abstract

The soybean pod borer (*Leguminivora glycinivorella* Matsumura) successfully survives the winter because of its high expression of 70-kDa heat shock proteins (HSP70s) during its overwintering diapause. The amount of HSP70s is different under different environmental stresses. In this study, inducible heat shock protein 70 and its constitutive heat shock cognate 70 were cloned by RT-PCR and RACE. These genes were named *Lg-hsp70* and *Lg-hsc70*, respectively. Gene transcription and protein expression after cold stress treatment (5°C to −5°C) were analyzed by western blotting and by qRT-PCR for four populations that were sampled in the northeast region of China, including Shenyang, Gongzhuling, Harbin and Heihe, when the soybean pod borer was in diapause. As the cold shock temperature decreased, the levels of Lg-HSP70s were significantly up-regulated. The amount of cold-induced Lg-HSP70s was highest in the southernmost population (Shenyang, 41°50′N) and lowest in the northernmost population (Heihe, 50°22′N). These results support the hypothesis that the soybean pod borer in the northeast region of China displays phenotypic plasticity, and the accumulation of Lg-HSP70s is a strategy for overcoming environmental stress. These results also suggest that the induction of HSP70 synthesis, which is a complex physiological adaptation, can evolve quickly and inherit stability.

## Introduction

Diapause is a strategy by which insects can escape death and gradually adapt to the environment in response to adverse environmental conditions [1]. Studying the molecular mechanisms of diapause is of great significance because of the important role of diapause in insect survival. Many studies have shown that environmental stress is harmful to insects, even those exposed to sub-zero temperatures over a short period, and that a strong relation between insect death and protein denaturation exists [2]. The expression of HSP70s (70-kDa Heat Shock Proteins) is a normal strategy used by insects to avoid damage arising from environmental stress [3], [4].

As molecular chaperones, HSP70s can recover the normal structure of denatured proteins to repair and protect the cell as well as increase the tolerance and resistance of insects [4], [5], [6]. HSP70s have a conserved structure and prevail in living organisms [7]. Depending on the expression style, these proteins are classified as inducible HSP70 (70-kDa Heat shock protein,HSP70) or constitutive HSC70 (Heat shock cognate protein, HSC70) [8], [9]. In general, the levels of HSP70 are low in normal conditions and increase upon environmental stress, such as high or low temperatures and exposure to heavy metals or microbial infection. HSC70 is stably expressed and only slightly increases upon environmental stress [10].

The expression level of HSP70s can be up-regulated by low temperature during insect diapause. This up-regulation has been observed in *Chilo suppressalis* and *Sesamia nonagrioides*, which belong to Lepidoptera [11], [12], in *Drosophila triauraria* and *Drosophila melanogaster*, which belong to Diptera [13], [14], and in *Leptinotarsa decemlineata*, which belongs to Coleoptera [15]. RNAi was used to study the effect of HSP70 expression on cold-stress resistance. Similar to conditions when HSP70 expression was inhibited, *Pyrrhocoris apterus* has a less intense response to cold resistance in diapause, and the pupae of *Sarcophaga crassipalpis* have feeble cold resistance [4], [16]. Therefore, we hypothesize that high HSP70 expression plays an important role in increasing insect resistance to cold stress.

HSP70s are an example of adaptive phenotypic plasticity providing inducible tolerance to the environment [17], [18], [19]. Phenotypic plasticity allows insects to react to different environments [20], [21]. This characteristic prevails in different organisms and represents an important aspect of adaptive evolution [22], [23], [24]. Phenotypic plasticity does not only increase cold stress resistance but also allows it to be stably inherited, playing an important role in insect evolution [25], [26], [27]. Because *hsp70* is a plasticity gene, understanding the function of *hsp70s* could supply a good theoretical foundation for understanding insect adaptability to the environment [28], [29], [30]. To date, the homologous genes of *hsp70s* in relation to phenotypic plasticity have been studied in *Rhagoletis cerasi*, *Calliphora vicina*, *Lymantria dispar* and *Melitaea cinxia* [19], [31], [32], [33]. However, the variation between conspecifics of different geographical populations has not been widely assessed [34].

The soybean pod borer (*Leguminivora glycinivorella* Matsumura) belongs to Lepidoptera: Olethreutidae and is one of the most important soybean pests in the world. Only one generation of *L. glycinivorella* lives in the northeast region of China, which is the main soybean production region, and its diapause stage lasts from October to July [35], [36]. *L. glycinivorella* can cause severe losses in soybean yield and quality; the loss rate is from 10% to 30% in a general year and up to 50% for more severe situations in a typical year [37], [38]. To date, research concerning *L. glycinivorella* has primarily focused on environmental ecology and physiology and has not focused on the genetic regulation of the diapause stage. Because a significant difference in climatic conditions exists in the vast, northeast region of China, we presumed that phenotypic plasticity occurred in different populations of *L. glycinivorella* in response to cold stress. In this study, we cloned *Lg-hsp70*s and analyzed their expression levels under cold stress induction (5°C to −5°C) to establish a foundation for understanding the molecular regulation of cold resistance and of the ecological adaptability of different populations of *L. glycinivorella*.

## Materials and Methods

### Materials

**Figure 1:**
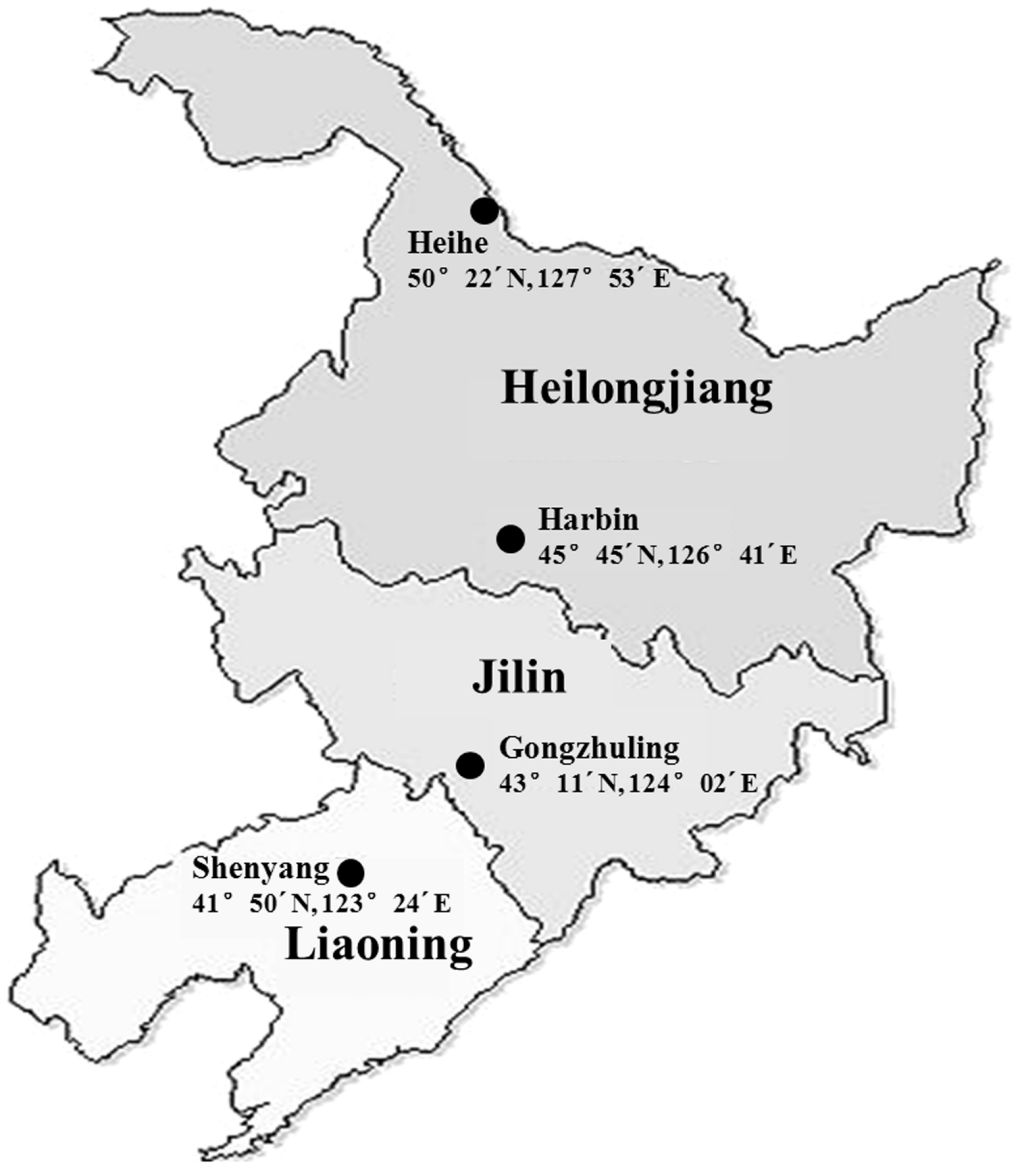
Collection sites of the soybean pod borer Northeast region map of China.

Insect collection sites: The soybean pod borer originated from four different populations: Harbin (Heilongjiang Province, 45°45′N, 126°41′E), Heihe (Heilongjiang Province, 50°22′N, 127°53′E), Gongzhuling (Jilin Province, 43°11′N, 124°02′E) and Shenyang (Liaoning Province, 41°50′N, 123°24′E). The total distance between the south and north populations is approximately 1182 km, and the linear distance from the north to south was approximately 700 km (Figure 1). For our study, we assumed that there was geographical isolation between the different collection sites. The average monthly temperature and the temperature range during diapause are shown in Table 1.

**Table 1:** Minimum and maximum mean air temperatures and their average from the nearest weather stations to the collection location for the soybean pod borer.

Collection site	Coordinates	October-June (°C)*		
		Min	Max	Mean
**Heihe**	50°22′N, 127°53′E	−22.6	16.2	−5.2
**Harbin**	45°45′N, 126°41′E	−19.4	18.9	−1.7
**Gongzhuling**	43°11′N, 124°02′E	−15.3	20.4	1.6
**Shenyang**	41°50′N, 123°24′E	−12.0	21.5	3.1

Note: ^*^ represents the air temperatures. Overwinter temperatures below ground typically correlate with air temperatures but are slightly higher (Xu *et al*, 1965).

Insect feeding: The adult insects were harvested in soybean fields in late July and reared in a glass on fresh soybean pods, with 20 adults in one glass. The female:male ratio was 1∶1. The glass was placed in a RXZ climate chamber (Ningbo, Jiangsu, China) at 25°C, with a light:dark cycle of 14 h∶10 h. The adults were removed after 12 h, and the eggs were harvested and transferred into a new glass with a soybean pod. After hatching, the larvae were reared to the fourth instar stage in the soybean pod and then harvested in the soybean pod. To induce diapause, the samples were placed in a sterilized soil box in a climate chamber with a mean temperature of 17°C. The temperature was 20°C during the 14-h photophase and 13°C during the 6 h of darkness. The lights were dimmed gradually over 2 h before and after the photophase, imitating sunrise and sunset. After the larvae became cocoons, we transferred the cocoons into a 5°C chamber, where the cocoons overwintered [39]. To diminish the impact of maternal effects, the fourth instar larvae used in the experiment were the second laboratory generation that was collected during the second consecutive year. To test the levels of the Lg-HSP70s, one set consisted of individuals that hatched from eggs collected during the same year that the experiment was performed.

### Methods

#### Cold shock treatment

The experiment began when the fourth instar larvae of the Heihe population were in the diapause midpoint (150 days). For the cold shock treatment, all fourth instar larvae were exposed to 5°C to −5°C for 1.5 h and then recovered at room temperature (25°C) for 1.5 h (none of the individuals died). Then, the larvae were frozen in liquid nitrogen and stored at −80°C. To determine the constitutive level of HSP70s, the control was a fourth instar larvae maintained at 25°C.

#### Cloning of *Lg-hsp70* and of *Lg-hsc70*

Total RNA isolation and first cDNA strand synthesis: RNA was extracted using an RNA isolation kit (Omega, USA), and the concentration and purity were determined using a NanoDrop spectrophotometer (NanoDrop 8000, Parallels, USA) and agarose gel electrophoresis. Then, first-strand cDNA synthesis was performed using a reverse transcription kit (ToYoBo, Japan).

Primer design and gene cloning: Based on the sequences of *hsp70* and *hsc70* from other insects in the GenBank database, degenerate primers for the conserved region of the gene family were designed using Primer Premier 5.0 software. The primer sequences are listed in Table 2. The PCR reaction for the conserved region was as follows: 3 min at 94°C; 35 cycles of 30 s at 94°C, 30 s at 57.8°C for *Lg-hsp70* or 30 s at 58.0°C for *Lg-hsc70*, 1 min at 72°C; and 10 min at 72°C. Then, the amplified fragment was isolated on a 1.0% agarose gel and ligated into the pMD18-T vector for sequencing. Based on the sequenced fragments of *Lg-hsp70* and *Lg-hsc70*, 5′ RACE and 3′ RACE primers were designed (see Table 2) and used following the manufacturer's protocol for the RACE kits. Full-length *Lg-hsp70* and *Lg-hsc70* were amplified using the same procedure and used for the following experiment.

**Table 2:** Primers used for the experiments in this study.

Primer type	Sequences of primers (5′–3′)	Use of primers
P1	GGCARGCCCHACVAARGATG	PCR for *Lg-hsp70*
P2	GTTGTCYTTBGTCATVGCTC	
P3	GAGGGMRTCGACTTCTAYACBTCCATCAC	PCR for Lg-hsc70
P4	CTTGTACTTCTCTGCNTCYTGBACCAT	
P5	GCGCACCTTTGCTGAGTTACTCTAC	For full-length *Lg-hsp70*
P6	CTGTCTTGTCTGAACGGAATGTTC	
P7	CTAAGCTGTTCATCTACTAGTTAAAACTACGACTGAAG	For full-length *Lg-hsc70*
P8	GATGGTGATTGCGTATGGAATGTTTAG	
5′PGSP1	CTCCTGCGGCGGACTTCACTTCG	5′ RACE for *Lg-hsp70*
5′PGSP2	TCTTTTGTGGCCTGTCTTTGTGAGTCGTTGAAGT	
5′CGSP1	GTTCACCATACGGTTGTCGAAGTC	5′ RACE for *Lg-hsc70*
5′CGSP2	GACACATCGAAGGTACCGCCGCCGAGATCG	
3′PGSP1	AGGTGCAGGACCTGCTGCTGCTCGA	3′ RACE for *Lghsp70*
3′PGSP2	ATCAACCCCGACGAGGCCGTCGCTTA	
3′CGSP1	CGGCAAGTTCGAGCTCACCGGCATC	3′ RACE for *Lg-hsc70*
3′CGSP2	GAAGGAGACCATCCAGGCCAAG	
*hsp70*F	CGTGTCCATCCTGACCATCG	qRT-PCR for *Lg-hsp70*
*hsp70*R	CAGTGCGCAGACGACGGAG	
*hsc70*F	GTCCAGGAGTTCAAGAGGAAGTACAAG	qRT-PCR for *Lg-hsc70*
*hsc70*R	GAAGAGAGAGTCGATTTCGATGCTAG	
*β-actin*F	GGCGACATAGCACAGCTTCTC	qRT-PCR for *β-actin*
*β-actin*R	ATCCTCCGTCTGGACTTGGC	

Bioinformatics software: The full-length sequences of *Lg-hsp70* and *Lg-hsc70* were analyzed using DNAStar software. The identity analysis was performed using the BLAST search program, which is available on the NCBI website (http://www.ncbi.nlm.gov/BLAST/). Multiple sequence alignment and phylogenetic analysis were performed using the ClustalW program (http://www.ebiac.uk/clustalw/) and the MEGA 4.1 software with the neighbor-joining method. The PSORT II program (http://psort.hgc.jp/form2.html) was used to calculate the nuclear localization signal fragment. The analyses of protein domains were performed using ProtParam (http://www.cn.expasy.org/tolls/ProtParam), ScanProsite (http://prosite.expasy.org/scanprosite/) and Swiss-Model (http://www.swissmodel.expasy.org/SWISS-MODEL.html) software tools.

#### Determining the total protein concentration

The fourth instar larvae were weighed after cold shock. Then, the total protein was isolated using a CytoBuster kit (Novagen, Germany), and the concentration of the protein was determined using a BCA kit (Thermo, USA). The isolated protein was diluted to 2.5 ng/µl in PBS and stored at −80°C. Then, 25 ng of each sample was separated by 12% SDS-PAGE (Mini-PROTEAN Tetra Cell, Bio-Rad). Each treatment was performed in 3 replicates, and β-actin (approximately 42 kDa) was used as a reference.

After SDS-PAGE, the sample was electroblotted onto a PVDF membrane and blocked in 5% dried skim milk.The membrane was incubated with a mouse monoclonal HSP70 antibody that could identify both HSP70 and HSC70 (H5147, Sigma, USA) (1∶5000, V:V) and with a mouse monoclonal β-actin antibody (1∶5000, V:V) (A5441, Sigma, USA) at 4°C overnight. After the membrane was washed three times in TBST for 10 min each, goat anti-mouse lgG (H+L) was used as the secondary antibody (Sigma, USA) (1∶1000, V:V) at room temperature for 2 h and then washed three times in TBST for 10 min each. The blots were developed in DAB and photographed. The bandwidth and gray level were analyzed using ImageJ 4.0 software. Then, a one-way ANOVA was computed using SPSS 16.0 software, with P<0.05.

#### Quantitative real-time PCR

qRT-PCR primers were designed using the *Lg-hsp70* and *Lg-hsc70* sequences (see Table 2). The products were 179 bp and 141 bp, respectively. Using *β-actin* as the reference gene, the primers were designed according to Li, and the amplified fragment was 132 bp [40]. Total RNA was extracted using an RNA isolation kit (Omega, USA), and the concentrations were brought to 100 ng/µl using DEPC-treated water in all samples. Then, cDNA synthesis was performed using a reverse transcription kit (ToYoBo, Japan).

Standard curve: Using the first-strand cDNA as a template and the above-mentioned primers for PCR amplification, the PCR products were purified using a Gel Extraction Kit (Omega, USA), ligated into the pMD18-T vector (TaKaRa, Japan) and transformed into *Escherichia coli* competent cells with blue-white color selection. Positive clones were harvested and cultured overnight. The plasmid was extracted and identified by PCR. Then, the positive clones were sequenced at Shanghai Sangon Biological Engineering Technology & Services Co., Ltd. (Shanghai, China). The correctly identified plasmid was quantified using a NanoDrop (NanoDrop 8000, Parallels, USA) and used at a 10x dilution as the standard for the qRT-PCR reaction. Each sample had 3 replicates. The standard equation was derived based on the critical number of cycles (threshold cycle, Ct) and on the logarithmic values of the initial template copy number. The qRT-PCR reaction used a fluorescent dye, SYBR Green I. The qRT-PCR reaction conditions were as follows: 95°C denaturation 30 s, 95°C denaturation 10 s, and 40 cycles of 60°C annealing and extension for 30 s. The Ct value of the reaction was read by the Opticon 3 software.

Data processing: The data were calculated using the 2^-ΔΔCt^ method after standard genetic homogenization treatment. For the control, the value of ΔΔCt was 0 (2^0^ = 1). Statistical significance was determined using a one-way ANOVA and post-hoc Duncan multiple range tests with the SPSS 16.0 software. The significance was established at P<0.05.

## Results

### Sequence analysis of *Lg-hsp70* and *Lg-hsc70*

**Figure 2:**
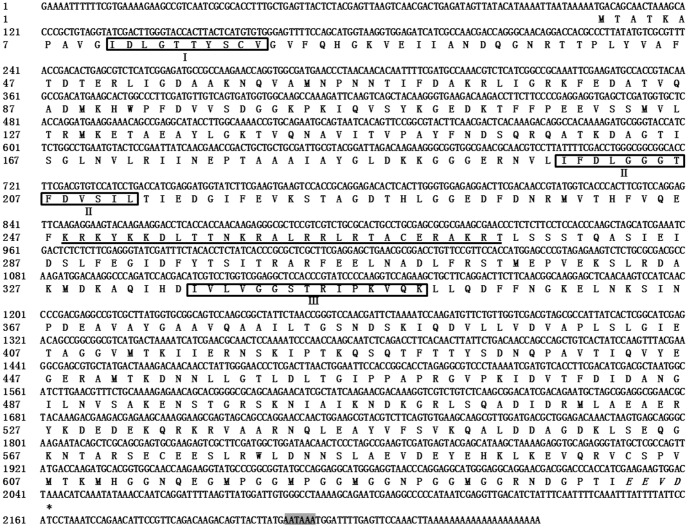
Nucleotide and deduced amino acid sequences of *Lg-hsp70*. The upper sequences indicate the nucleotides, and the lower sequences show the inferred amino acids. Start and stop codons are in bold. The signature sequences of the HSP70s family are shown in boxes, the nuclear localization signal sequence is underlined, the consensus sequence EEVD at the C-terminus is indicated in italics and the consensus polyadenylation signal (AATAAA) is shown in a gray box.

**Figure 3:**
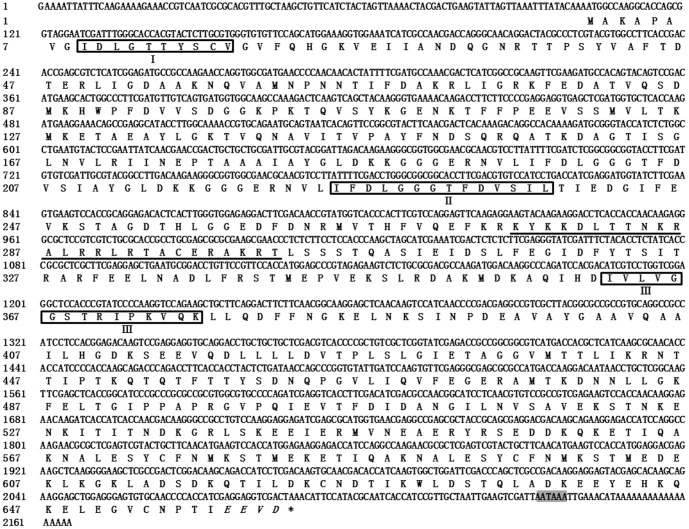
Nucleotide and deduced amino acid sequences of *Lg-hsc70*. The upper sequences indicate the nucleotides, and the lower sequences show the inferred amino acids. The start and stop codons are in bold. The signature sequences of the HSP70s family are shown in boxes, the nuclear localization signal sequence is underlined, the consensus sequence EEVD at the C-terminus is indicated in italics and the consensus polyadenylation signal (AATAAA) is shown in a gray box.

*Lg-hsp70* and *Lg-hsc70* were cloned and deposited into the GenBank database under accession numbers KF731995 and KC844150, respectively. The coding amino acids are shown in Figures 2 and 3. The full-length *Lg-hsp70* sequence was 2253 bp [excluding poly(A)] and included a 102-bp 5′-UTR, a 189-bp 3′-UTR, and a 1941-bp ORF, which encoded 646 aa. The molecular weight was 70.73 kDa, and the PI was 5.54. The positive charge of disability (Arg + Lys) was 83, and the negative charge of disability (Asp + Glu) was 93. In addition, the full-length *Lg-hsc70* sequence was 2147 bp [excluding poly(A)] and included a 102-bp 5′-UTR, a 59-bp 3′-UTR and a 1986-bp ORF, which encoded 661 aa. The molecular weight was 73.23 kDa, and the PI was 5.30. The positive charge of disability (Arg + Lys) was 76, and the negative charge of disability (Asp + Glu) was 101. The protein sequences showed that both Lg-HSP70 and Lg-HSC70 have 3 conserved HSP70 family domains, EEVD at the end of the C-terminus [41] and two putative bipartite nuclear localization signal (NLS) sequences (Figures 1 & 2).

### Phylogenetic analysis of Lg-HSP70 and Lg-HSC70

**Figure 4:**
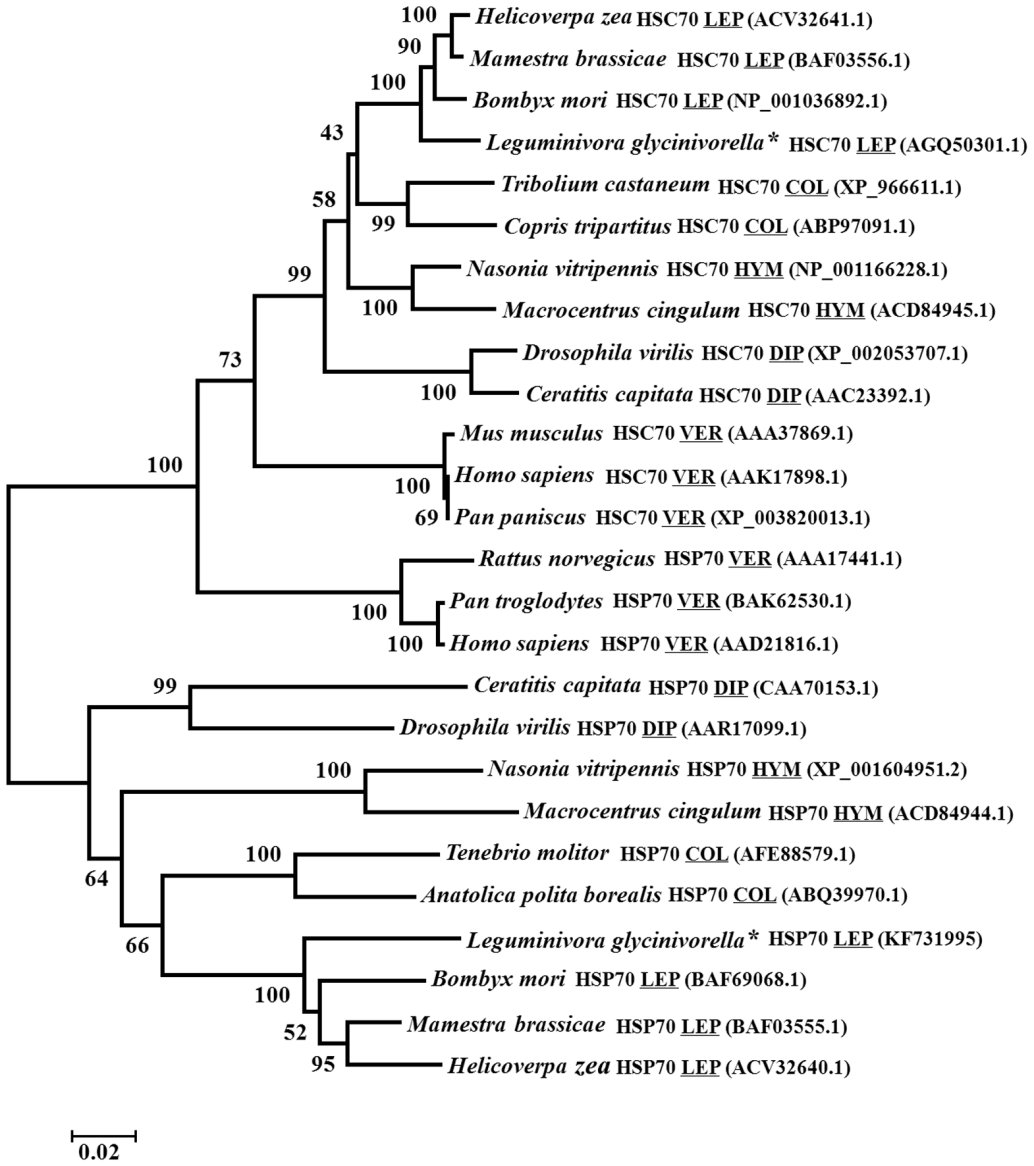
Phylogenetic tree of the HSP70 and HSC70 sequences, constructed using the neighbor-joining method. A three-letter code has been included to indicate the order name of the corresponding insects and vertebrates (COL =  Coleoptera, LEP =  Lepidoptera, DIP =  Diptera, HYM =  Hymenoptera and VER =  Vertebra). The values indicated on the branches correspond to bootstrap percentages (BP).

Using homology analysis, we found that Lg-HSP70 was more than 73.6% similarity with other known HSP70; in particular, it had 89.5% identity with *Bombyx mori* (BAF69068) and *Mamestra brassicae* (BAF03555) and 70.6% identity with vertebrates. Lg-HSC70 had more than 78.1% similarity with other known HSC70s, and it had 86% identity with *Helicoverpa zea* (ACV32641) and 72.6% identity with vertebrates. A phylogenetic tree was constructed, revealing that the HSP70 family could be divided into two main branches (Figure 4). One branch is HSC70, which also contains the other 11 HSC70 genes, and the other branch is HSP70, which contains all other HSP70 genes. Therefore, we could confirm that *Lg-hsc70* and *Lg-hsp70* are constitutive and inducible genes, respectively.

### Expression of the Lg-HSP70 genes

**Figure 5:**
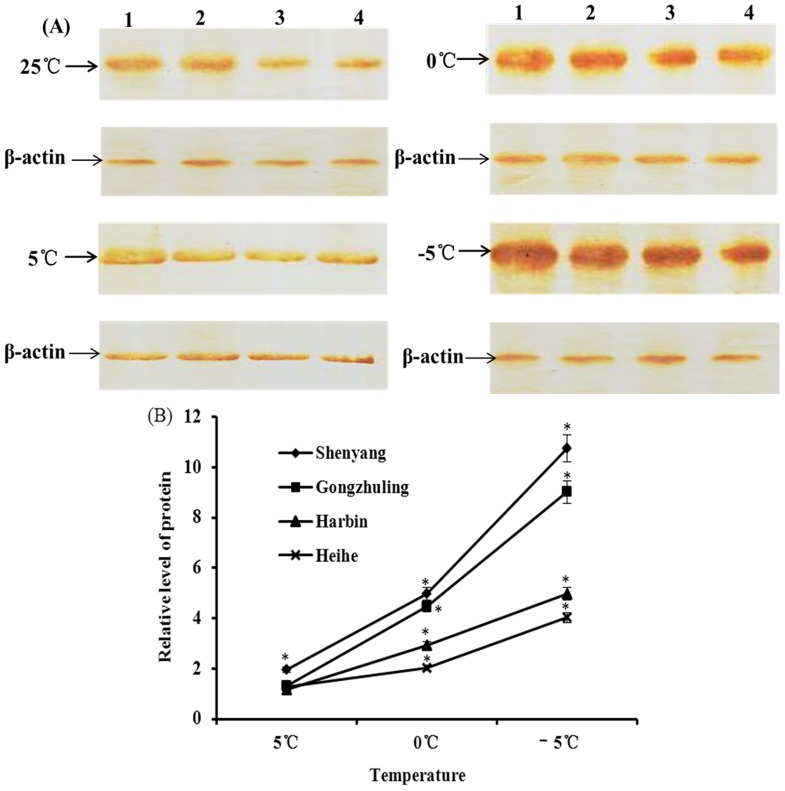
Relative analysis of Lg-HSP70s levels in different geographical populations after cold treatment. (A) Western blotting of Lg-HSP70s. 1: Shenyang, 2: Gongzhuling, 3: Harbin, 4: Heihe. (B) Relative gray levels of Lg-HSP70s (*: significant difference compared with control, P<0.05).

The transcription and expression of Lg-HSP70s were determined using qRT-PCR and western blotting, respectively. The results showed that the levels of the Lg-HSP70s were not significantly different (P>0.05) between field- and laboratory-derived populations in the four cities. Three factors were considered to analyze the relationships among temperature (cold stress and control), sex (female and male) and population (50°22′N, 45°45′N, 43°11′N and 41°50′N). However, only the treatment between temperature and population had a significant effect on the Lg-HSP70 levels (P<0.05). Western blotting showed that the expression of Lg-HSP70s that originated from the same geographical population was significantly up-regulated as the temperature decreased, and it peaked at -5°C (Figure 5). Compared with the amount of Lg-HSP70s at the same temperature, there was a significant increasing trend as the latitude decreased. Shenyang (41°50′N) expressed the most Lg-HSP70s, and Heihe (50°22′N) expressed the least Lg-HSP70s. Because no differences in the HSP70 level were observed, we concluded that the laboratory-rearing conditions had little effect on the HSP70 level. These results revealed that cold stress has a significant inducible effect on the expression of Lg-HSP70s, indicating that phenotypic plasticity variation for Lg-HSP70s occurs in different geographical populations.

qRT-PCR analysis: The positive clones were identified by PCR. The soybean pod borer cDNA sequences for *Lg-hsp70*, *Lg-hsc70* and *β-actin* had 100% homology, indicating that the recombinant plasmid contained the target fragment. Using a UV spectrophotometer, the standard concentrations of *Lg-hsp70*, *Lg-hsc70* and *β-actin* were found to be 22 ng/µl, 18 ng/µl and 15 ng/µl, respectively. The copy numbers of the *Lg-hsp70*, *Lg-hsc70* and *β-actin* standards were 7.01×10^9^, 5.79×10^9^ and 4.87×10^9^ copies/µl, respectively. The equations of the standard curves of *Lg-hsp70*, *Lg-hsc70* and *β-actin* were Y = -3.124X+40.601, Y = -3.124X+46.327 and Y = -3.297X+39.876, respectively, where Y represents the value of Ct, and X represents the logarithmic value of the template. The correlation coefficients were R^2^ = 0.992, R^2^ = 0.998 and R^2^ = 0.997, respectively. When the template cDNA was diluted, the Ct value was up-regulated 3 to 4 cycles, and the concentration decreased with each 10-fold dilution. The slopes of three standard curves were near the expected value of -3.322, with a correlation coefficient close to 1.000, suggesting that the results of the qRT-PCR could be used for the quantitative detection of *Lg-hsp70* and *Lg-hsc70* over a wide range.

Expression of *Lg-hsp70* induced by cold shock: Compared with the control (25°C), the expression level of *Lg-hsp70* could be induced in the four populations at 5°C. *Lg-hsp70* increased 7.08 fold in Shenyang, which was significantly higher than the levels in the other populations, and there was no significant difference among the other three populations. At 0°C, the *Lg-hsp70* transcripts were present at a high level in each population, and there was a significant difference in their levels compared with the level of the control (P<0.05). The expression of *Lg-hsp70* in each population reached its peak at –5°C, increasing by 80.18 fold, 69.32 fold, 41.37 fold and 25.01 fold in Shenyang, Gongzhuling, Harbin and Heihe, respectively, compared with the control. A significant difference existed among the different geographical populations (P<0.05) (see Table 3). Thus, cold treatment had a significant effect on inducing *Lg-hsp70* at diapause.

**Table 3:** Differences in *Lg-hsp70* and *Lg-hsc70* levels induced by cold stress temperatures in the soybean pod borer.

Temperature (°C)	Induced level of *Lg-hsp70*				Induced level of *Lg-hsc70*			
	Shenyang	Gongzhuling	Harbin	Heihe	Shenyang	Gongzhuling	Harbin	Heihe
−5°C	80.18±0.54a	69.32±0.11b	41.37±0.92c	25.01±0.45d	7.91±0.07a	5.09±0.14b	4.67±0.82c	2.07±0.06d
0°C	26.74±0.04a	20.02±0.79b	13.02±0.03c	9.57±0.25d	3.51±0.53a	2.44±0.06b	2.21±0.46b	1.62±0.47c
5°C	7.08±0.12a	5.58±0.17b	5.02±0.01b	5.34±0.37b	1.25±0.31a	1.21±0.52a	1.06±0.73a	0.93±0.46a

Note: The different letters above the bars (a–d) were the result of a multi-comparison, which indicated that the means are significantly different (P<0.05).

Expression of *Lg-hsc70* induced by cold shock: There was a small, inducible effect on the transcription of *Lg-hsc70* by cold treatment from −5°C to 5°C, and the level was significantly lower than that of *Lg-hsp70*. At 5°C, the expression level of *Lg-hsc70* did not differ from the control. At 0°C, the expression level of *Lg-hsc70* significantly increased (P<0.05); however, there was no significant difference between Gongzhuling and Harbin (P>0.05). The highest transcription of *Lg-hsc70* was observed at −5°C, increasing by 7.91 fold, 5.09 fold, 4.67 fold and 2.07 fold in Shenyang, Gongzhuling, Harbin and Heihe, respectively, compared with the control. A significant difference existed among the four geographical populations (see Table 3).

## Discussion

The Lg-HSP70 and Lg-HSC70 sequences both contain a conserved amino acid site, and the consensus sequence EEVD at the end of the C-terminus revealed that the two proteins belong to the DanK cytoplasmic-type protein of the HSP70 family. In addition, two nuclear localization sites (NLS) that play important roles in the inhibitory effect of HSP70s against nucleolar impairment induced by oxidative stress were identified [42].

To determine whether *Lg-hsp70* and *Lg-hsc70* were inducible or constitutive, a cluster analysis was performed. The results showed that *Lg-hsp70* and *Lg-hsc70* belong to two different branches of *hsp70* and *hsc70*, respectively. Some recent studies have reported that the expression level of *hsp70* increases significantly after induction by environmental stress but that *hsc70* is expressed with a time lag and with a low range compared with *hsp70* [43]. In our experiment, the expression time and rate of increase of *Lg-hsp70* significantly differed from that of *Lg-hsc70*, which supports the conclusion that *Lg-hsp70* is an inducible gene and that *Lg-hsc70* is a constitutive gene. The difference in expression of the two genes may be because the *Lg-hsp70* gene has no intron and *Lg-hsc70* does, which affects the split joint of the mRNA [44], [45].

Selection for low-temperature stress: Although *L. glycinivorella* overwinters in buried soil, this insect can still be exposed to harmful environmental temperatures. In northeastern China, the depth of the frozen soil varies with year and location [46]. Consequently, the optimum overwinter depth for *L. glycinivorella* is unpredictable, and exposure to the cold is possible. According to the temperature data for frozen soil from the northeastern region and the report that −5°C was the lethal temperature for *L. glycinivorella* to overwinter [47], −5°C and −10°C were designed as the pretreatment temperatures in this study. The results showed that all larvae were dead at −10°C; therefore, −5°C was selected as the treatment temperature.

Lg-HSP70 protein expression and mRNA transcription were analyzed after cold stress in the four geographical populations. The results revealed that HSP70s had a strong relation with insect resistance to cold stress [11], [34]. Thus, this mechanism is most likely a strategy that *L. glycinivorella* uses to react to cold stress. Additionally, we found that the up-regulation of Lg-HSP70s varies with latitude. A southern population may have less chance of experiencing cold stress during their overwintering diapause compared with a northern population [48]. Some studies have shown that the production of HSP70s as a response to temperature shock is less intense in organisms that are more frequently exposed to unfavorable temperatures in their habitat compared with those organisms exposed to benign conditions [49]. Research concerning *Leptinotarsa decemlineata* showed the same trend when comparing a southern population (Bonin, Poland, 54°N) to a northern population (Lodeynoye Pole, Russia, 60°N) [50].

Because HSP70 production requires more energy, this production may incur significant costs to the synthesis of other proteins [51], [52]. Therefore, the northern population might have evolved other more reasonable, less costly mechanisms to cope with cold stress [48], [53]. In recent years, possible mechanisms have been studied and include accumulating low-molecular-weight cryoprotectants [54], synthesizing antifreeze proteins [55] and remodeling the structure of the cell membrane [56]. In contrast, with the development of molecular biology, the function, reactions and location of HSP70s also could be identified by bioinformatics tools and proteomics techniques, such as 2-dimensional electrophoresis and mass spectra which have become powerful tools to discover and validate protein-coding genes. Some proteomic studies showed that different isoforms of the HSP70 family, including HSP68, HSP70Ba, and HSP70Bb, exist and that several of them displayed strong increases in protein abundance during environmental variations [57], [58]. Furthermore, the gene annotation and localization might play a pivotal role to facilitate identification and analysis of HSP70s in the integrated genome of *L. glycinivorella*. Thus, on the basis of this research, application of proteomic and genomic strategies could supply good support for the relevant mechanism of *L. glycinivorella* from different populations.

*L. glycinivorella* from different populations showed a differential response to cold stress. The significant differences in the expression levels of LgHSP70s in the southern (Shenyang, 41°50′N) and northern populations (Heihe, 50°22′N) showed strong evidence for phenotypic plasticity among the four different populations [59]. The phenotypic plasticity in these populations contributes to the soybean pod borer response to different selective pressures under different environmental conditions and has great potential for improving their ability to resist environmental stress [60], [61]. Because relative reports of the damage of *L. glycinivorella* date to before the 1950s [38], we assume that there must be a long history of *L. glycinivorella* damaging the soybean crop in northeastern China. Although no reports of genetic variability can be found, we believe that phenotypic plasticity variation based on epigenetic mechanisms has occurred for a long period in different geographic populations of *L. glycinivorella* so that they could adapt to the different ecological conditions. Thus, because this variation can be stably inherited, it will undoubtedly have a profound effect on population adaptability [62], [63], [64].
